# Global Microbiome Diversity Scaling in Hot Springs With DAR (Diversity-Area Relationship) Profiles

**DOI:** 10.3389/fmicb.2019.00118

**Published:** 2019-02-22

**Authors:** Lianwei Li, Zhanshan (Sam) Ma

**Affiliations:** ^1^Computational Biology and Medical Ecology Lab, State Key Laboratory of Genetic Resources and Evolution, Kunming Institute of Zoology, Chinese Academy of Sciences, Kunming, China; ^2^Kunming College of Life Sciences, University of Chinese Academy of Sciences, Kunming, China; ^3^Center for Excellence in Animal Evolution and Genetics, Chinese Academy of Sciences, Kunming, China

**Keywords:** biogeography of hot-spring microbiome, DAR (diversity-area relationship), MAD (maximal accrual diversity), local to regional (global) diversity (LED/LGD), biogeographic differences between archaea and bacteria

## Abstract

The spatial distribution of biodiversity (i.e., the biogeography) of the hot-spring microbiome is critical for understanding the microbial ecosystems in hot springs. We investigated the microbiome diversity scaling (changes) over space by analyzing the diversity-area relationship (DAR), which is an extension to classic SAR (species-area relationship) law in biogeography. We built DAR models for archaea and bacteria with 16S-rRNA sequencing datasets from 165 hot springs globally. From the DAR models, we sketch out the biogeographic maps of hot-spring microbiomes by constructing: (i) DAR profile—measuring the archaea or bacteria diversity scaling over space (areas); (ii) PDO (pair-wise diversity overlap or similarity) profile—estimating the PDO between two hot springs; (iii) MAD (maximal accrual diversity) profile—predicting the global MAD; (iv) LRD/LGD (ratio of local diversity to regional or global diversity) profile. We further investigated the differences between archaea and bacteria in their biogeographic maps. For example, the comparison of DAR-profile maps revealed that the archaea diversity is more heterogeneous (i.e., more diverse) or scaling faster than the bacterial diversity does in terms of species numbers (species richness), but is less heterogeneous (i.e., less diverse) or scaling slower than bacteria when the diversity (Hill numbers) were weighted in favor of more abundant dominant species. When the diversity is weighted equally in terms of species abundances, archaea, and bacteria are equally heterogeneous over space or scaling at the same rate. Finally, unified DAR models (maps) were built with the combined datasets of archaea and bacteria.

## Introduction

Hot springs are one of the extreme environments on the earth planet. Hot spring microbiomes play a critical role in shaping the geothermal ecosystems. The structures and functions of microbial communities inhabiting hot springs have their somewhat unique characteristics compared with non-geothermal environment microbiomes (Inskeep et al., 2013a,b; Song et al., 2013; Masaki et al., 2016; Poddar and Das, 2018), such as soil microbiome (Fierer et al., 2012; Hartmann et al., 2014), marine microbiome (Gajigan et al., 2018), and human microbiome (Huttenhower et al., 2012). Hot springs are often abundant in thermophilic, hyperthermophilic, and thermoresistant bacterial and archaeal taxa (Urbieta et al., 2014a,b). For example, the hot springs located in the Chilas and Hunza areas of Pakistan with 90–95°C temperature harbor abundant phylum *Thermotogae* (Amin et al., 2017). Many reports have suggested that hot spring microbial communities are extremely heterogeneous and are often dominated by the thermophilic bacterium (e.g., Cole et al., 2013; Sharp et al., 2014; Masaki et al., 2016; Poddar and Das, 2018). Even though some recent studies showed that microbial eukaryotes, especially microbes from the phyla of *Ascomycota* and *Basidiomycota*, may also be important components of hot springs microbiomes (de Oliveira et al., 2015; Salano et al., 2017; Liu et al., 2018; Oliverio et al., 2018), the microbial communities routinely consist of Bacteria and Archaea (Meyer-Dombard and Amend, 2014; Hedlund et al., 2015; Merkel et al., 2017).

The stability of hot spring environments is routinely determined by the steady state of their microbial diversity in a specific environment, where water temperature, pH, and chemical composition are often the most important factors to influence the diversity (Mohanrao et al., 2016; Amin et al., 2017; Chan et al., 2017; Ghilamicael et al., 2017; Poddar and Das, 2018). In general, there is an inversely proportional relationship between microbial diversity and temperature of hot spring (Cole et al., 2013; De León et al., 2013; Amin et al., 2017; Chan et al., 2017). In addition to temperature, water pH is another primary environmental factor directly influencing microbial diversity in hot springs (Inskeep et al., 2013a,b; Xie et al., 2015). The water pH is determined by the chemical composition in hot springs. The role of chemical composition in designing the structure of geothermal microbial communities should not be underestimated, which sometimes play together with the pH (Jiang et al., 2015; Geesey et al., 2016). In spite of the extensive studies on the microbial diversities in hot springs, which suggest that the biodiversity of microbial communities vary with physicochemical conditions and biogeographical location of inhabiting hot springs, to the best of our knowledge, the hot-spring microbiome diversity scaling on regional or global scales from a biogeography perspective has not been investigated yet.

To investigate the microbiome diversity scaling on regional/global scale, one of the most powerful theoretical tools is the classic species-area relationship (SAR) power law, which achieved a rare “law” status in ecology and biogeography. The SAR is often described with a power function *S* = *cA*^*z*^, where *S* is the number of species accumulated from a region of size A, and *z* is termed species (number) scaling parameter. The study of SAR can be traced back to the nineteenth century (Watson, 1835; Arrhenius, 1921; Preston, 1960, 1962) and the relationship was said to inspire MacArthur and Wilson (1967) to establish their island biogeography theory, which helped to shift the focus of ecology from population level to community level.

Theoretically, a series of extensions of the classic SAR to general diversity-area relationship (DAR) were introduced by the author's group recently (Ma, 2018a,b). The extensions were justified to remedy a limitation with classic SAR, where the biodiversity is measured with the number of species or the so-termed *species richness*. Species richness can be a rather meaningful measure for biodiversity in the case of large plants and animals, but in many other cases (especially for microbes), it is a poor measure of biodiversity because it ignores the differences in species abundances. For example, 1,000 of panda and one billion of panda will weigh in the same with species richness; but if the latter number were the case, panda would not have been on the endangered species list. The DAR extension was facilitated by the adoption of the Hill numbers (Hill, 1973; Chao et al., 2012, 2014) as general diversity measures, which weight diversity differently depending on the so-termed diversity order. In terms of the Hill numbers, biodiversity can be measured by the so-termed diversity profile (Chao et al., 2012, 2014), which calculates a series of Hill numbers, weighted differently by the species abundance distribution (SAD). Therefore, Hill numbers are now well-recognized as the most appropriate measures for alpha-diversity and its multiplicative partition of beta-diversity is also considered with advantages over other beta-diversity measures. With the new DAR approach, four sets of new tools: DAR profile, PDO (pair-wise diversity overlap) profile, and MAD (maximal accrual diversity) profile, and LRR/LGR [local to regional (global) diversity ratio], can be established with the parameters from DAR modeling. These profiles, together with DAR models offer powerful tools, not only for quantifying the regional/global scaling of biodiversity, but also for sketching out the biogeography maps of biodiversity distribution (Ma, 2018a,b). In the present study, we apply the DAR approach to analyzing the global biodiversity scaling of hot-spring microbiome by reanalyzing the 16S-rRNA marker gene abundance datasets of 165 hot springs on a global scale, previously collected and published by Sharp et al. (2014). We further sketch out and compare the biogeography “maps” of archaea and bacteria, and highlight their differences in biodiversity distribution.

The four profiles, DAR (diversity-area relationship), PDO (pair-wise diversity overlap), MAD (maximal accrual diversity) profile, LRD (local to regional diversity ratio), we build for archaea, bacteria, and their combined assemblages offer tools to sketch out the biogeography maps with different themes. While, the map theme profiled by DAR is the diversity scaling (difference or heterogeneity) over space, the theme profiled by PDO is the similarity of diversity over space. While the map theme profiled by MAD is the theoretically maximal accrual diversity (essentially the maximal gamma diversity), the theme profiled by LRD/LGD is then the local *vs*. regional diversity comparison, which answers a simple question: how much, on average, a local sample can represent the regional or global diversity.

## Materials and Methods

### The Hot Spring Microbiome Datasets

The datasets of 165 hot-spring microbiome were originally collected and reported by Sharp et al. (2014). Their 16S-rRNA OTU (operational taxonomic unit) tables were generated from 165 microbiome samples taken from sediment, soil, and mat in Western Canada and Taupo Volcanic Zone, New Zealand (Sharp et al., 2014). A total of 1,162,553 high quality sequences were obtained from the 165 samples with 634–15,283 sequences per sample. There were 61,910 OTUs, including 7,964 archaea and 53,946 bacteria, when those sequences were clustered at the 97% identity threshold. Further information on the datasets is referred to Sharp et al. (2014).

### Definitions and Computational Procedures

**Figure 1:**
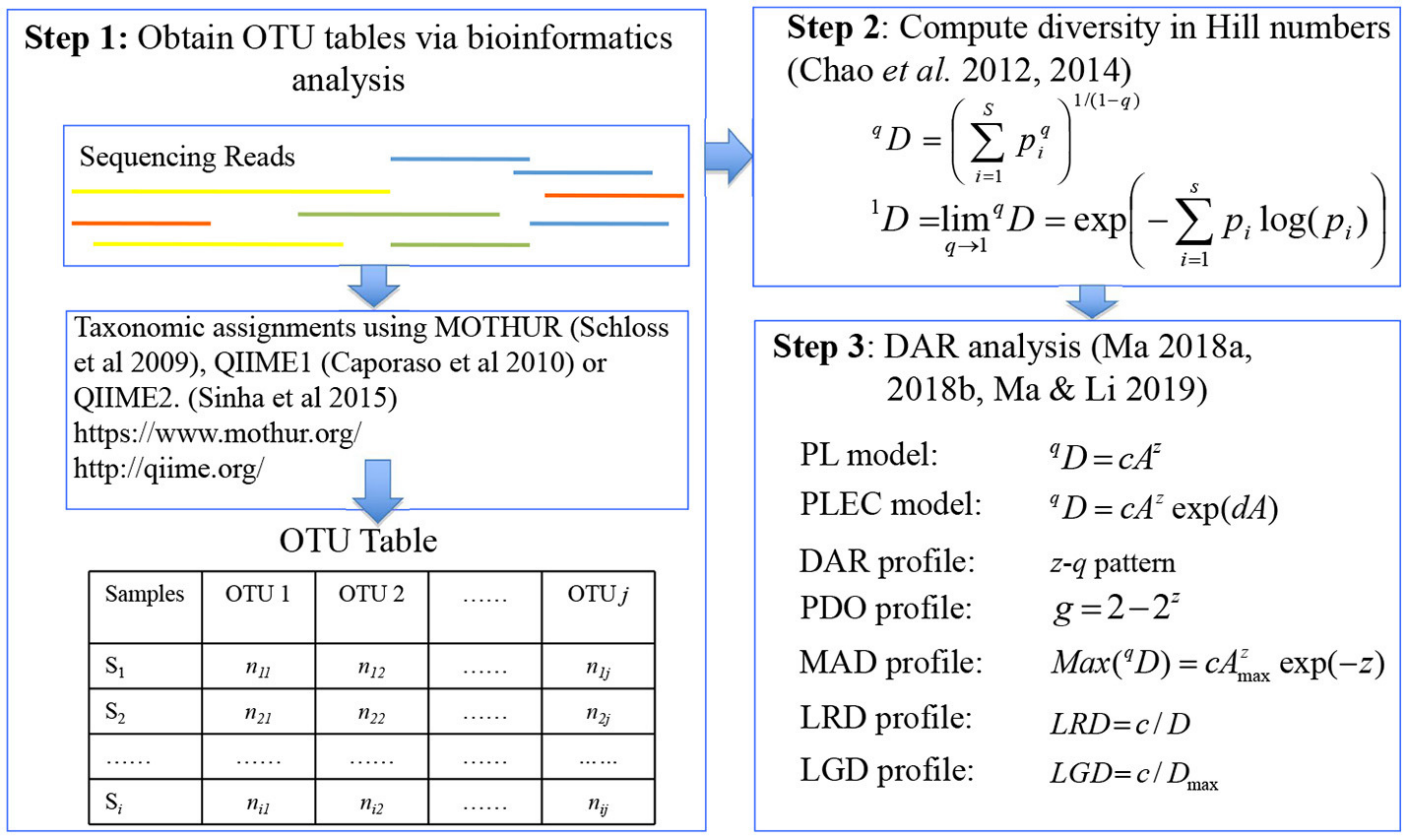
A diagram showing the three steps involved in DAR (diversity-area relationship) analysis: major software packages (MOTHUR, QIIME, QIIME2), data format (OTU table), definitions (Hill numbers), models (PL and PLEC), and concepts (profiles) were illustrated.

Three steps are involved in building DAR models with microbiome datasets (see Figure 1): (i) bioinformatics analysis of 16S-rRNA data to get OTU tables (e.g., Schloss et al., 2009; Caporaso et al., 2010; Sinha et al., 2015). The microbiome quality control project: baseline study design and future directions. Genome Biology. Vol. 16: 276, https://doi.org/10.1186/s13059-015-0841-8; (ii) computing species or OTU diversities measured with the Hill numbers (Chao et al., 2012, 2014; Ma, 2017); (iii) constructing the DAR models (Ma, 2018a,b).

### Diversity Measured in Hill Numbers

The Hill numbers (Hill, 1973; Jost, 2007; Chao et al., 2012) are considered as the most appropriate measure for alpha diversity,


(1) $$\begin{array}{l} {\;}^{q}D={\left(\overset{S}{\underset{i=1}{\sum }}p_{i}^{q}\right)}^{1/\left(1-q\right)} \end{array}$$

where *S* is the number of species, *p*_i_ is the relative abundance of species *i, q* is the order number of diversity. The Hill number is not defined when *q* = 1, but its limit as *q* approaches to *1* exists in the following form:


(2) $$\begin{array}{l} {\;}^{1}D={\underset{q\to 1}{\lim}}^{q}D=\exp\left(-\overset{s}{\underset{i=1}{\sum }}p_{i}\log\left(p_{1}\right)\right) \end{array}$$

The parameter *q* determines the sensitivity of the Hill number to the relative frequencies of species abundances. If *q* = 0, the species abundances do not weigh at all and ^0^*D* = *S*, which is simply the species richness. When *q* = 1, ^1^*D* equal the *exponential* of Shannon entropy, and is interpreted as the number of typical or common species in the community because ^1^*D* is weighted proportionally by species abundances. When *q* = 2, ^2^*D* equal the reciprocal of Simpson index, i.e., the number of *dominant* or very abundant species in the community (Chao et al., 2012) because ^2^*D* is weighted in favor of more abundant species. The general interpretation of ^*q*^*D* (diversity of order *q*) is that the community has a diversity of order *q*, which is equivalent to the diversity of a community with ^*q*^*D* = *x* equally abundant species. The so-termed diversity profile refers to the Hill numbers at different diversity order *q* (Jost, 2007; Chao et al., 2012, 2014).

### The DAR Models and DAR Profile

Ma (2018a) extended SAR (species area relationship) to general DAR (diversity area relationship), in which diversity is measured with Hill numbers. The first DAR model, which borrowed the same power law (PL) function from the classic SAR, is:


(3) $$\begin{array}{l} {\;}^{q}D=cA^{z} \end{array}$$

where ^*q*^*D* is diversity measured in the *q*-*th* order Hill numbers, *A* is *area*, and *c* and *z* are parameters.

A second DAR model is the power law with exponential cutoff (PLEC) model, which was originally introduced to SAR modeling by Plotkin et al. (2000), Ulrich and Buszko (2003), and Tjørve (2009) is:


(4) $$\begin{array}{l} {\;}^{q}D=cA^{z}\exp\left(dA\right), \end{array}$$

where *d* is a third parameter and is usually negative in the DAR models, and exp(*dA*) is the exponential decay term, which eventually overwhelms the power law behavior at very large value of *A*.

The following log-linear transformed equations can be used to estimate the parameters of the DAR models:


(5) $$\begin{array}{l} \text{ln}\left(D\right)=\text{ln}\left(c\right)+z\text{ ln}\left(A\right) \end{array}$$

(6) $$\begin{array}{l} \text{ln}\left(D\right)=\text{ln}\left(c\right)+z\text{ ln}\left(A\right)+dA \end{array}$$

The linear correlation coefficient (*R*) and *p*-value are used to judge the goodness of the model fitting.

Ma (2018a) defined the relationship between DAR-PL (power law) model parameter (*z*) and diversity order (*q*), or *z-q* trend, as the *DAR profile*, which comprehensively describes the change of diversity scaling parameter (*z*) with the diversity order (*q*).

### Predicting MAD (Maximal Accrual Diversity) With PLEC-DAR Models

Ma (2018a) derived the maximal accrual diversity (MAD) in a cohort or population based on PLEC model [Equations (4) and (6)] as follows:


(7) $$\begin{array}{l} Max{(}^{q}D){=}^{q}D_{\max}=c{\left(-\frac{z}{d}\right)}^{z}\text{exp}\left(-z\right)=cA_{\max}^{z}\text{exp}\left(-z\right) \end{array}$$

where *A*_max_ is the number of areas accrued to reach the maximum and is equal to:


(8) $$\begin{array}{l} A_{\max}=-z/d \end{array}$$

and all parameters are the same as in Equations (4) and (6).

Similar to the previous definition for DAR profile (*z-q* pattern), Ma (2018a) defined the MAD profile (*D*_max_-*q* pattern) as a series of *D*_max_ values corresponding to different diversity order (*q*).

### Pair-Wise Diversity Overlap (PDO) Profile

The *pair-wise diversity overlap* (*g*) of two bordering areas of the same size (i.e., the proportion of the new diversity in the second area) is:


(9) $$\begin{array}{l} g=2-2^{z} \end{array}$$

where *z* is the scaling parameter of DAR-PL model [Equations (3) and (5)]. If *z* = 1, then *g* = 0 and there is no overlap (similarity); and if *z* = 0, then *g* = 1 and there is a total overlap. In reality, *g* should be between 0 and 1. Since *g* is between 0 and 1, one may even use percentage notation to measure PDO.

Similar to previous definitions for DAR profile (*z-q* pattern) and MAD profile (*D*_max_-*q* pattern), Ma (2018a) defined the PDO profile (*g-q* pattern) as a series of PDO values corresponding to different diversity order (*q*).

### The Ratio of Local Diversity to Regional (or Global) Accrual Diversity

Ma and Li (2019) defined the LRD (or LGD) as the ratio of local diversity of an *averaged area* to the *regional diversity* accrued or the global MAD (maximal accrual diversity). The dividend (local diversity) is ideally estimated with the parameter *c* of the DAR-PL model, but can be approximated with the parameter *c* of the DAR-PLEC model. The divisor can be either regional accrual diversity (which can be estimated with PLEC model directly) or global maximal accrual diversity (which is simply the MAD or *D*_*max*_). Hence, in general, two similar metrics can be defined, depending on the regional or global scale is adopted: one is the ratio of local diversity to regional diversity (LRD), and another is the ratio of local diversity to global MAD (LGD). The LRD (LGD) can be computed with the following formulae, respectively:


(10a) $$\begin{array}{l} LRD=c/D \end{array}$$

(10b) $$\begin{array}{l} LGD=c/D_{\max} \end{array}$$

where *D* can *D*_*max*_ be computed with the PLEC model directly (Equations 4 and 7, respectively), *c* can be estimated or approximately with the PL or PLEC model. The LRD (LGD) at different diversity orders (*q* = 0, 1, 2, …) were defined as LRD (LGD) profile, or local to regional (local to global) diversity scaling profile (Ma and Li, 2019). It is essentially the ratio of alpha to gamma diversity.

### Re-sampling Procedure to Enhance the Robustness of DAR Modeling

The accumulation order of areas in DAR modeling may influence the estimation of parameter *c* in fitting PL/PLEC models (Equations 3–6). When there is not a natural spatial sequence (or arrangement) among the communities sampled, or the arrangement information is not available, arbitrarily choosing an accumulation order (arrangement) can be problematic. To avoid the potential bias from an arbitrary order of the hot spring microbiome samples, we totally permutated the orders of all the community samples under investigation, and then randomly choose 100 orders of the communities generated from the permutation operation. In other words, rather than taking a single arbitrary order for accruing community samples in one-time fitting to the DAR model, we iteratively perform the DAR model-fitting 100 times with the 100 randomly chosen orders. Finally, the averages of the model parameters from the 100 times of DAR fittings are adopted as the model parameters of the DAR. In the case of this study, we do not have detailed information on the geographic locations of the hot-spring microbiome samples, the re-sampling scheme is adopted to remedy the deficiency.

## Results and Discussion

### DAR Analysis of the Archaea

Table 1 displays the parameters from fitting the DAR (diversity-area relationship) PL (power law) and PLEC (power law with exponential cutoff) models to the datasets of archaea in hot springs. The *p*-values in Table 1 show statistically significant fitting (in all models *p* < 0.001) of both PL and PLEC to the datasets. The PLEC model has an advantage of being able to estimate the MAD (maximal accrual diversity) or *D*_*max*_, which essentially measures the accrued diversity in a population or cohort, with the so-termed MAD-profile, as explained previously. The other two parameters from PL model, scaling parameter (*z*) and pair-wise diversity overlap (PDO) parameter (*g*) define the DAR-profile and PDO profile, respectively. From Table 1, we summarized the following findings:

- The archaea diversity scaling (changes across space) fitted to the PL model successfully, with *p* < 0.001, and succeeded in all 100 times of re-sampling. It also fitted to PLEC model successfully *p* < 0.001, but with slightly less robustness given that some failures out of the 100 times of re-sampling occurred, especially at the high diversity orders. This does not imply that PLEC model is not applicable to the archaea DAR at all; it only suggests that the order of accruing areas (hot spring samples) influences the fitting of the model.
- The DAR profile: the parameter *z-q* series from the PL model is: *z-q* = {0.867[*q* = 0], 0.575[*q* = 1], 0.512[*q* = 2], 0.492[*q* = 3]}, a monotonically decreasing trend with the increase of diversity order (q). This indicates that at lower diversity orders, the spatial heterogeneity is larger than that at higher orders. The highest heterogeneity is at the species richness level (*q* = 0). Since at higher diversity order, the dominant species were given more weights in computing the diversity (Hill numbers). This suggests that the hot spring microbiomes are more homogenous (less heterogeneous or less diverse) in terms of their dominant species. Since at lower diversity order, the rare species were given more weights in computing the diversity (Hill numbers). This suggests that the hot spring microbiomes are more heterogeneous (more diverse) in terms of rare species.
- The PDO profile: the parameter *g-q* series from the PL model is: *g-q* = {0.170[*q* = 0], 0.505[*q* = 1], 0.568[*q* = 2], 0.588[*q* = 3]}, a monotonically increasing trend with the increase of diversity order (*q*). This indicates that at lower diversity orders, the pair-wise diversity overlap (similarity) is smaller than that at higher orders. The lowest diversity overlap (similarity) is at the species richness level (*q* = 0), and the highest similarity occurred at the diversity order *q* = 3. This finding further supports the finding revealed by the DAR-profile in the previous item (ii).
- The MAD profile: the parameter *D*_*max*_-*q* series estimated from the PLEC model is: *D*_*max*_-*q* = {8,397[*q* = 0], 248.3[*q* = 1], 68.8[*q* = 2], 45.0[*q* = 3]}, shows a monotonically decreasing trend with the increase of diversity order (*q*). This, of course, is determined by the nature of the diversity (Hill numbers). The *D*_*max*_(*q* = 0) = 8,397 is simply the maximal accrual of species richness since the Hill numbers at *q* = 0 is the species richness. For example, at diversity order *q* = 1, the Hill numbers (our measure for the diversity) are equivalent to (function of) Shannon diversity index. This suggests that the maximal accrual diversity in terms of the Shannon index is 248.3. Similar interpretations can be made for *q* = 2, and 3. The maximal accrual species richness of 8,397 means that, it needs to accrue *A*_max_ = 262 hot spring sites (areas) globally to reach this theoretical asymptote of species richness in the hot spring microbiome.
- There appears a trend of decreasing correlation coefficient (*R*) with increasing diversity order (*q*). This should be expected because with increasing *q*, the complexity associated with non-linearity in higher order entropy (i.e., Hill numbers) is increased. Consequently, goodness-of-fitting to the linear models (Equations 5, 6) is likely to decline.

**Table 1:** The parameters of DAR (diversity-area relationship) for the ***Archea*** in the hot springs, computed with 100 times of re-sampling from the totally permutated hot springs.

**Diversity order and statistics**		**Power Law (PL)**						**PL with Exponential Cutoff (PLEC)**							
		***z***	**ln(*c*)**	***R***	***p*-value**	***g***	N^*^	***z***	***d***	**ln(*c*)**	***R***	***p*-value**	N^*^	***A_***max***_***	***D_***max***_***
*q* = 0	Mean	0.867	4.657	0.983	<0.001	0.170	100	1.058	−0.004	4.206	0.990	<0.001	90	262	8396.9
	Std. Err.	0.012	0.054	0.001	<0.001	0.015		0.024	0.000	0.080	0.001	<0.001			
	Min	0.650	2.804	0.942	<0.001	−0.408		0.666	−0.014	1.368	0.958	<0.001			
	Max	1.268	5.690	0.997	<0.001	0.430		1.900	0.000	5.565	0.998	<0.001			
*q* = 1	Mean	0.575	2.771	0.923	<0.001	0.505	100	0.836	−0.006	2.201	0.951	<0.001	81	143	248.3
	Std. Err.	0.013	0.063	0.005	<0.001	0.014		0.025	0.000	0.082	0.003	<0.001			
	Min	0.234	0.882	0.799	<0.001	0.095		0.359	−0.017	0.115	0.847	<0.001			
	Max	0.930	4.278	0.987	<0.001	0.824		1.482	0.000	3.899	0.989	<0.001			
*q* = 2	Mean	0.512	1.740	0.900	<0.001	0.568	100	0.724	−0.005	1.293	0.931	<0.001	71	157	68.8
	Std. Err.	0.013	0.064	0.007	<0.001	0.013		0.025	0.000	0.084	0.005	<0.001			
	Min	0.172	−0.033	0.604	<0.001	0.185		0.317	−0.017	-0.302	0.713	<0.001			
	Max	0.860	3.193	0.980	<0.001	0.873		1.341	0.000	2.919	0.983	<0.001			
*q* = 3	Mean	0.492	1.392	0.893	<0.001	0.588	100	0.675	−0.004	1.042	0.924	<0.001	64	163	45.0
	Std. Err.	0.013	0.061	0.007	<0.001	0.013		0.026	0.000	0.085	0.007	<0.001			
	Min	0.152	−0.208	0.535	<0.001	0.240		0.244	−0.016	-0.454	0.652	<0.001			
	Max	0.815	2.805	0.980	<0.001	0.889		1.289	0.000	2.676	0.989	<0.001			

N* = the number of successful fitting to DAR model from 100 times of random re-sampling of the individual orders. All the parameters were averaged from the N^*^ times of re-sampling, i.e., the parameters from successful fitting. Detailed parameters of the 100 DAR models from the 100 times of re-sampling are provided in the online Supplementary Tables (Tables S1–S6).

### DAR Analysis of the Bacteria

We did the same DAR analysis with bacteria dataset, and the results are exhibited in Table 2. We further performed Wilcox non-parametric significance test of the differences between Archaea and Bacteria in their DAR parameters, and it turned out that (i) regarding the PL-model, archaea-DAR and bacteria-DAR have significantly different DAR parameter values except for the diversity order *q* = 1. (ii) Regarding the PLEC model, archaea-DAR, and bacteria-DAR have significantly different DAR parameter values at the higher diversity orders (*q* = 2, 3), but no significant differences occurred at the lower diversity orders (*q* = 0, 1). These test results justify our attempt to separately build DAR models for archaea and bacteria. Since the format of Tables 1, 2 are exactly the same, our explanations for the bacteria-DAR is presented relatively brief intentionally. From Table 2, we summarized the following findings:

- The bacteria diversity scaling (changes across space) fitted to both the PL and PLEC models successfully, with all *p* < 0.001. The goodness-of-fitting is equally well with that for the archaea DAR models in the previous sub-section.
- The DAR profile: the parameter *z-q* series from the PL model is: *z-q* = {0.830[*q* = 0], 0.616[*q* = 1], 0.575[*q* = 2], 0.544[*q* = 3]}, a monotonically decreasing trend with the increase of diversity order (*q*). This indicates that at lower diversity orders, the spatial heterogeneity is larger than that at higher orders. The highest heterogeneity is at the species richness level (*q* = 0). This pattern *per se* is the same as that of archaea-DAR.
  If we further compare both the DAR profiles (see statistical tests in Table 3), we found that archaea has a larger diversity scaling parameter (*z-*value*s*) at diversity order *q* = 0 (i.e., species richness, but smaller scaling parameter (z-values) at diversity order *q* = 2 or 3. At diversity order *q* = 1, which is equivalent to the diversity measured with Shannon entropy and weighs all species in proportion with their relative abundance levels, archaea and bacteria showed no significant difference in their scaling parameter (*z*-values). These findings indicate that archaea is more heterogeneous or scaling faster than bacteria does in terms of species numbers (species richness), but is less heterogeneous or scaling slower than bacteria when the diversity (Hill numbers) were weighted computationally in favor of more abundant dominant species. When the diversity (Hill numbers) is weighted equally in terms of species abundances, archaea, and bacteria are equally heterogeneous over space or scaling at the same rate.
  The above finding also highlighted the necessity of using Hill numbers as general diversity measures (the diversity profile) over using a single *ad-hoc* diversity measure such as Shannon entropy or Simpson's index, because the latter may lead to inconsistent results or loss of information. This also shows the necessity of using DAR profile, a series of scaling parameter values (z) across different diversity orders (*q*), rather than using a single scaling parameter as in classic SAR (species-area relationship) analysis.
- The PDO profile: the parameter *g-q* series from the PL model is: *g-q* = {0.217[*q* = 0], 0.456[*q* = 1], 0.502[*q* = 2], 0.535[*q* = 3]}, a monotonically increasing trend with the increase of diversity order (*q*). This pattern is the same as that of the archaea DAR PDO profile.
  Similar to the previous DAR-profile comparison between archaea and bacteria, the archaea has a smaller PDO overlap (similarity) than the bacteria has at species richness level (*q* = 0), but has a larger PDO overlap (similarity) at the higher diversity order *q* = 2 or 3. At the diversity order *q* = 1, archaea and bacteria have the same level of diversity overlap (similarity) across space. The interpretation for this finding is exactly the same as that for the DAR-profile above.
- The MAD profile: the parameter *D*_*max*_-*q* series estimated from the PLEC model is: *D*_*max*_-*q* = {55,489[*q* = 0], 2,831.1[*q* = 1], 427.7[*q* = 2], 207.9 [*q* = 3]}, showing a monotonically decreasing with the increase of diversity order (*q*). This, of course, is determined by the nature of the diversity (Hill numbers). The *D*_*max*_(*q* = 0) = 55,489 is simply the maximal accrual of species richness since the Hill numbers at *q* = 0 is the species richness. Similar interpretations can be made for *q* = 1, 2, and 3. The maximal accrual species richness of 55,489 means that, it needs to accrue *A*_max_ = 256 hot spring sites (areas) globally to reach this theoretical asymptote of species richness in the hot spring microbiome.
- Similar to the previous DAR models for archaea, there appears a trend of decreasing correlation coefficient (*R*) with increasing diversity order (*q*). This should be expected as explained previously.

**Table 2:** The parameters of DAR (diversity-area relationship) for the ***Bacteria*** in the hot springs, computed with 100 times of re-sampling from the totally permutated hot springs.

**Diversity Order and Statistics**		**Power Law (PL)**						**PL with Exponential Cutoff (PLEC)**							
		***z***	**ln(*c*)**	***R***	***p*-value**	***g***	N^*^	***z***	***d***	**ln(*c*)**	***R***	***p*-value**	N^*^	***A_***max***_***	***D_***max***_***
*q = 0*	Mean	0.830	6.738	0.983	<0.001	0.217	100	1.031	−0.004	6.241	0.989	<0.001	88	256	55489.3
	Std. Err.	0.011	0.051	0.002	<0.001	0.014		0.022	0.000	0.073	0.001	<0.001			
	Min	0.568	5.634	0.930	<0.001	−0.091		0.674	−0.011	4.814	0.959	<0.001			
	Max	1.064	7.985	0.998	<0.001	0.517		1.444	0.000	7.517	0.999	<0.001			
*q = 1*	Mean	0.616	5.016	0.929	<0.001	0.456	100	0.914	−0.006	4.317	0.959	<0.001	87	144	2831.1
	Std. Err.	0.018	0.084	0.005	<0.001	0.020		0.033	0.000	0.115	0.003	<0.001			
	Min	0.262	2.907	0.691	<0.001	−0.104		0.389	−0.018	1.805	0.777	<0.001			
	Max	1.073	6.671	0.987	<0.001	0.801		1.607	0.000	6.301	0.997	<0.001			
*q = 2*	Mean	0.575	3.283	0.906	<0.001	0.502	100	0.827	−0.005	2.733	0.945	<0.001	73	152	427.7
	Std. Err.	0.015	0.075	0.007	<0.001	0.016		0.027	0.000	0.099	0.006	<0.001			
	Min	0.223	1.122	0.609	<0.001	0.019		0.398	−0.014	1.058	0.709	<0.001			
	Max	0.987	4.982	0.982	<0.001	0.833		1.391	0.000	4.566	0.997	<0.001			
*q = 3*	Mean	0.544	2.697	0.892	<0.001	0.535	100	0.777	−0.005	2.209	0.935	<0.001	70	152	207.9
	Std. Err.	0.014	0.067	0.008	<0.001	0.014		0.025	0.000	0.088	0.007	<0.001			
	Min	0.193	0.776	0.504	<0.001	0.132		0.410	−0.013	0.787	0.694	<0.001			
	Max	0.901	4.382	0.985	<0.001	0.857		1.288	0.000	3.847	0.991	<0.001			

N* = the number of successful fitting to DAR model from 100 times of random re-sampling of the individual orders. All the parameters were averaged from the N^*^ times of re-sampling, i.e., the parameters from successful fitting. Detailed parameters of the 100 DAR models from the 100 times of re-sampling are provided in the online Supplementary Tables (Tables S1–S6).

**Table 3:** The *p*-values of Wilcox non-parametric significance test between the differences between Archaea and Bacteria in their DAR parameters.

**Diversity order (*q*)**	**DAR-PL model**						**DAR-PLEC model**		
	***z***			***g***			***z***		
	**≠**	**>**	**<**	**≠**	**>**	**<**	**≠**	**>**	**<**
*q =* 0	0.030	0.015	0.985	0.030	0.985	0.015	0.245	0.123	0.878
*q* = 1	0.137	0.932	0.069	0.137	0.069	0.932	0.114	0.943	0.057
*q* = 2	0.005	0.997	0.003	0.005	0.003	0.997	0.032	0.984	0.016
*q* = 3	0.011	0.995	0.005	0.011	0.005	0.995	0.028	0.986	0.014

While the above *pattern* for bacteria MAD-profile is similar to the pattern for archaea MAD profile, the vis-a-vis comparison of both the MAD profiles is a different story. Obviously, the values of bacteria *D*_*max*_ are far larger than the values of archaea *D*_*max*_. Indeed, the difference is consistent with biological (ecological) reality that there are far more bacteria species than archaea species in hot springs. Unfortunately, unlike the cases of DAR and PDO profiles, we cannot perform the permutation (randomization) test for MAD-profile (*D*_*max*_). This is because the *D*_*max*_ was computed based on the average parameter values form 100 times of re-sampling. We believe biological (ecological) observations justify our claims that MAD-profiles are also different between the archaea and bacteria.

### DAR Analysis With the Combined Datasets of Archaea and Bacteria

Since there are significant differences between the archaea and bacteria in their DAR parameters, ideally, independent DAR models should be built for each kingdom. However, there is no doubt that they cohabitate (coexist) in the hot spring environment. Therefore, building unified DAR models (Table S7) for the combined archaea and bacteria is justified. Table S7 shows that the DAR models fitted to the combined datasets of archaea and bacteria equally well with those for the archaea or bacteria, independently. For practical purpose such as conservation planning, the unified models (Table S7) are obviously more convenient, but for theoretical (mechanistic) inquiries, the separately built DAR models previously (Tables 1, 2) should be more appropriate. Since the pattern of the unified DAR models are similar to the separately built ones, except some nuances, which make little differences for practical applications. As to the theoretical implications of those nuances, we recommend the use of those separately built DAR models directly. Therefore, we do not further compare the subtle differences between the unified and separate DAR models here.

### The Ratio of Local Diversity to Regional (or Global) Accrual Diversity

The LRD (or LGD) is defined as the ratio of the local diversity of an *averaged area* to the regional diversity [or the global maximal accrual diversity (MAD)]. The dividend (local diversity) can be estimated with parameter *c* of the DAR-PL model, and the divisor can be either regional accrual diversity (which can be estimated with PLEC model directly) or global maximal accrual diversity (which is simply the MAD or *D*_*max*_). Note that we defined LRD/LDG at different diversity orders (*q* = 0, 1, 2, 3, …) as LRD/LGD profile.

Here we only computed LGD, the global version of the ratio. Table 4 listed the LGD for the archaea, bacteria and their combined microbiome, at each diversity order (*q*). For example, at species richness level (*q* = 0), the LGD is between 1.24 and 1.57%, which suggests that, on average, a single (local) hot spring only hosts between 1.24 and 1.57% of the global scale diversity. At high diversity orders, the ratios increased (up to 9% approximately). Another interesting observation is that at the species richness (*q* = 0), the LGD for archaea is lower than that for bacteria. However, at higher diversity orders (*q* = 1, 2, 3), the trend is reversed.

**Table 4:** The LGD (the ratio of local diversity to global maximal accrual diversity) profile for the archaea, bacteria, and combined communities in the hot springs.

**Diversity order (*q*)**	**Alpha-LGD** **(%) (Archaea)**	**Alpha-LGD** **(%) (Bacteria)**	**Alpha-LGD** **(%) (Combined)**
*q =* 0	1.25	1.52	1.57
*q* = 1	6.43	5.33	5.75
*q* = 2	8.29	6.23	5.95
*q* = 3	8.95	7.13	6.12

## Discussion

With the gold rush of microbial community ecology, thanks to the revolutionary metagenomic sequencing technology, the classic SAR has been called for new missions. Green et al. (2004) and Horner-Devin et al. (2004) published, in the same issue of the journal *Nature*, the first two studies on the SAR of microbes. The following year, two other important studies by Bell et al. (2005) and Smith et al. (2005) were published in two other leading journals, *Science* and *PNAS*, respectively. The SAR power law exponent (*b*) values from those studies were 0.074 (fungi), 0.019–0.040 (bacteria in marsh sediment), 0.26 (bacteria in tree holes), and 0.134 (phytoplankton). According to Green and Bohannan (2006) review, the reported SAR exponents in microbes were in the range between 0.019 and 0.470, but most values were below 0.2 (8 out of 11 studies). A major limitation of these pioneering studies on the testing of SAR with microbes is then low throughput of DNA sequencing technology in detecting bacteria, and consequently the diversity and SAR exponent were significantly underestimated. Even with the technology limitation, the reported exponent values have already indicated the applicability of SAR in microbes, and recent studies further confirmed the validity of microbial SAR (e.g., Noguez et al., 2005; Peay et al., 2007; Bell, 2010; Barreto et al., 2014; Pop Ristova et al., 2014; Ruff et al., 2015; Terrat et al., 2015; Várbíró et al., 2017). For example, nearly a decade after Green and Bohannan (2006) review, the range of exponent (*z*) of microbial SAR is nearly unchanged and most studies have still been limited to bacteria and archaea (Barreto et al., 2014).

While the classic SAR has been well-recognized as one of the most significant laws in ecology and biogeography, it is not without limitations. The recent extension from SAR to DAR by Ma (2018a,b) generalized the scaling law of biodiversity from species richness (the number of species) to general diversity measures (the Hill numbers). Furthermore, DAR profile, PDO profile, MAD profile, LRD/LGD profile based on DAR models can offer useful novel tools to sketch out the biogeography maps, which comprehensively characterize the biodiversity scaling over space and time.

Despite the large number studies of microbial SAR in various environments, to the best of our knowledge, the SAR of the hot-spring microbiome has not been reported in existing literature. In consideration of the more general nature of DAR over SAR, we skipped SAR and directly applied DAR modeling to reanalyze the hot spring microbiome datasets of Sharp et al. (2014). In fact, our DAR analysis, as presented in previous sections, included SAR as a special case when diversity order *q* = 0. Our study therefore provides the first glimpse of the SAR/DAR of the hot spring microbiome. The results and conclusions we obtained should certainly be further verified in future with more extensive datasets of the hot spring microbiomes. Although the sample size of 165 hot springs, we used, is not small, the future studies should attempt to collect samples from more diverse regions from different continents to validate our study on a truly global scale.

## Author Contributions

ZM designed the study and wrote the paper. LL performed the data analysis. All authors approved the submission.

### Conflict of Interest Statement

The authors declare that the research was conducted in the absence of any commercial or financial relationships that could be construed as a potential conflict of interest.
